# Sudden unexpected death: a national programme which will establish genetic testing and cardiological screening of families in the UK

**DOI:** 10.1007/s00292-022-01143-8

**Published:** 2022-12-07

**Authors:** Mary N. Sheppard

**Affiliations:** Department of Cardiovascular Pathology, St Georges Medical School, Jenner Wing, Cranmer Terrace London SW17 ORE, University of London, Cranmer Terrace, London, SW17 ORE UK

**Keywords:** Sudden cardiac death, Cardiac arrest, Genetic testing, Molecular autopsy testing, Cardiac examination

## Abstract

Sudden cardiac death (SCD) in young people is due to genetic cardiac causes in the majority of cases. In UK all cases have an autopsy and results are given to the family. We now have  a national pathway with detailed programme to establish that all members of the family  are screened by cardiologists in inherited cardiac centres . Also genetic testing of material taken at autopsy will be carried out in all cases which will be a valuable addition to the pathological and clinical findings. All this will aid the family in coming to terms with the tragic death and provide genotype phenotype correlation within the family to prevent future deaths.

Sudden unexpected death (SUD) is defined as natural unexpected death in witnessed cases as an acute event with time to death being < 1 h and in unwitnessed cases as person last seen alive < 24 h before being found dead [1]. It is also labelled as sudden cardiac death (SCD), as most of the causes are cardiac. SCD is frequent in older age groups due largely to ischaemic heart disease [2]. In younger people it is mainly due to sudden arrhythmogenic death syndrome (SADS) where the heart is morphologically normal and electrical abnormalities are the cause (cardiac channelopathies) and due to structural cardiomyopathies (CM). Both of these have mainly genetic causes. It is especially important that SCD in younger people is investigated by autopsy, as these genetic cardiac causes have important implications for their families [3–5].

There are many difficulties facing the pathologist in such cases in view of the genetic implications and prevention of further deaths within families, as we have published on previously [6]. There are four major issues arising.

1. Retention of cardiac tissue with storage and location of the retained tissue.
2. Consent to be obtained from the family to retain the tissue
3. The appropriate tissue to be taken for genetic testing, the pathway for the genetic testing and who should pay for this.
4. Who would arrange cardiological screening of the families?

Following this meeting, a UK charity funded by bereaved families called Cardiac Risk in the Young (CRY) established with me a nationally available free service for prospective reporting on the cardiac pathology of SCD. This work enables us to process hearts referred from coroners and pathologists from throughout the UK and we have built up over 7000 cases in this database, with the majority of cases due to SADS and CM (Fig. 1). Since 2013, we have collected post-mortem genetic material providing valuable positive results [7, 8].

**Fig. 1:**
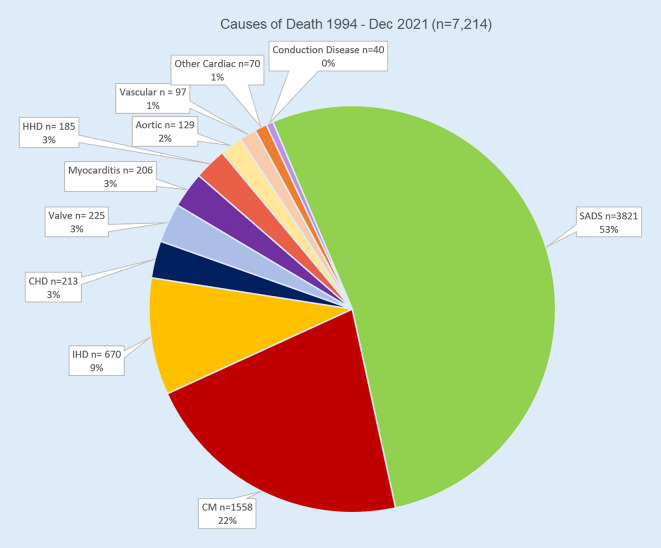
Causes of sudden cardiac deaths in database of 7000 cases within UK. *HHD* hypertensive heart disease, *IHD* Ischaemic heart disease, *CM* Cardiomyopathies, *CHD* Congenital heart disease

In the 21st century, the National Health Service (NHS) England sees its role as harnessing the power of genomic technology and science to improve the health of the population. In 2019, an NHS document on genomics and prevention resulted in the implementation of genetic medical service alliances in 2021 to deliver a single national testing directory covering use of all technologies from single genes to whole-genome sequencing. Most importantly from our point of view, it established a national genomic testing directory for rare and inherited cardiac diseases including aortopathies, vasculopathies, cardiac channelopathies and cardiomyopathies [9, 10]. This led to the establishment of seven genomic hubs in the UK (Fig. 2). These now provide the opportunity for systematic introduction of post-mortem genetic testing for SUD with cardiac causes. The Royal College of Pathologists has issued guidelines on the taking of genetic material at such autopsies, which was recently updated [11].

**Fig. 2:**
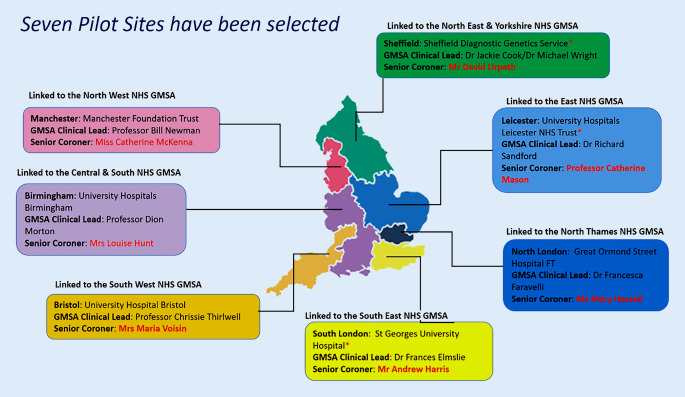
Seven pilot sites have been selected

With NHS England and charities including the British Heart Foundation (BHF) and CRY, I have worked on a pilot study to establish a multidisciplinary team of coroners, coroner officers, pathologists, cardiologists, geneticists and genetic scientists in each of the seven pilot sites. A coordinator has been appointed in each region to direct the pathway from the pathologist, to coroner, coroner officer, retention of tissue, taking of genetic sample, through to the family and referral to inherited cardiac conditions services within each genomic region. Genetic testing will be undertaken within the genetic hubs once the family has been seen by a specialist cardiologist and the cardiac phenotype has been established by the pathologist at autopsy. The logistics of tissue retention and sample handling for genetic testing will be supervised by the regional coordinator and will be appropriately funded.

The pilot study highlights the need for specialist cardiac pathology centres, as a study we undertook highlighted the need for this expertise [12]. This is needed to review the cardiac cause of death as cardiac phenotype is so important for family screening. As in oncology services, review is needed especially for challenging cases [13]. Yes, pathologists can get it right first time [14], but more training is needed in cardiac pathology as general training in autopsy does not prepare for the more challenging cardiomyopathies and congenital heart lesions [15]. We also need pathological guidelines for these entities, which we are undertaking within the European Association of Cardiovascular Pathology.

The autopsy rate for SUD in Europe varies widely, with an average of 43% and genetic testing in 48%, which leaves a large gap [16]. This pilot study, in which there should be an autopsy rate of 100%, will yield invaluable information to expand our knowledge of cardiac causes of death with clinical follow-up and genetic testing. The pathologist will be a vital part of the multidisciplinary team investigating these SUDS. This pilot development within the NHS represents a major breakthrough, as pathologists and coroners will no longer have to worry about costs and will have a coordinator to arrange the logistics and follow-up in each case. This should lead to full investigation and prevention of further deaths in families, as the majority of cases, especially in the young, are genetic.

## Declarations

This study has ethical approval within St Georges Medical School.

The supplement containing this article is not sponsored by industry.
